# Remote Assessment of Parkinson’s Disease Symptom Severity Using the Simulated Cellular Mobile Telephone Network

**DOI:** 10.1109/ACCESS.2021.3050524

**Published:** 2021-01-11

**Authors:** Athanasios Tsanas, Max A. Little, Lorraine O. Ramig

**Affiliations:** 1 Edinburgh Medical SchoolUsher Institute, The University of Edinburgh 3124 EdinburghEH16 4UXU.K.; 2 School of Computer ScienceUniversity of Birmingham 1724 BirminghamB15 2TTU.K.; 3 Department of Speech, Language, and Hearing ScienceUniversity of Colorado Boulder 1877 BoulderCO80309USA; 4 National Center for Voice and Speech 497057 DenverCO80014USA

**Keywords:** Decision support tool, Parkinson’s disease, nonlinear speech signal processing, telemedicine

## Abstract

Telemonitoring of Parkinson’s Disease (PD) has attracted considerable research interest because of its potential to make a lasting, positive impact on the life of patients and their carers. Purpose-built devices have been developed that record various signals which can be associated with average PD symptom severity, as quantified on standard clinical metrics such as the Unified Parkinson’s Disease Rating Scale (UPDRS). Speech signals are particularly promising in this regard, because they can be easily recorded without the use of expensive, dedicated hardware. Previous studies have demonstrated replication of UPDRS to within less than 2 points of a clinical raters’ assessment of symptom severity, using high-quality speech signals collected using dedicated telemonitoring hardware. Here, we investigate the potential of using the standard voice-over-GSM (2G) or UMTS (3G) cellular mobile telephone networks for PD telemonitoring, networks that, together, have greater than 5 billion subscribers worldwide. We test the robustness of this approach using a simulated noisy mobile communication network over which speech signals are transmitted, and approximately 6000 recordings from 42 PD subjects. We show that UPDRS can be estimated to within less than 3.5 points difference from the clinical raters’ assessment, which is clinically useful given that the inter-rater variability for UPDRS can be as high as 4–5 UPDRS points. This provides compelling evidence that the existing voice telephone network has potential towards facilitating inexpensive, mass-scale PD symptom telemonitoring applications.

## I. Introduction

Parkinson’s Disease (PD) is a chronic neurodegenerative disorder characterized by the progressive deterioration of motor function as well as the emergence of considerable non-motor problems [1]. The PD incidence rate is approximately 20/100,000 [2] and the prevalence rate exceeds 100/100,000 [3]; moreover it is believed that an additional 20% of people with Parkinson’s (PWP) might be undiagnosed [4]. Early PD stages are mainly characterized by three hallmark symptoms: *bradykinesia* (slow and reduced amplitude of movement), *rigidity* (resistance to passive movement), and *tremor* (while at rest) [5].

Medication and surgical intervention can alleviate some of the symptoms and improve quality of life for most PWP [6], although there is currently no known cure. To optimize treatment, PWP are typically followed up by expert clinical staff at relatively sparse (six to twelve month) intervals. Unfortunately, this contemporary triage of symptom management likely underestimates the true fluctuation of symptom severity. More regular PD symptom assessment would be of considerable benefit, for example, to optimize treatment regimes, but this is not possible given the available resources and the established assessment setting which requires the physical presence of PWP in the clinic.

In current clinical practice, medical raters physically examine PWP and map symptom severity on appropriate clinical scales (metrics). The Unified Parkinson’s Disease Rating Scale (UPDRS) [7] is the most widely used clinical metric for quantifying PD impairment [8], and attempts to quantify the full breadth of possible motor (muscle), non-motor PD symptoms, and complications of dopamine replacement therapies. The motor symptoms are quantified using the *motor-UPDRS*, which is a subset and highly correlated with the *total UPDRS* [9]. The motor-UPDRS ranges between 0 and 108, where 0 denotes healthy state, and 108 severe disabilities, and the total-UPDRS lies in the range between 0 and 176. In addition to UPDRS, the Hoehn and Yahr (H&Y) scale is often used, and it is possible to infer H&Y from UPDRS [10], [11]. Other clinical scales are sometimes used in some medical centers, but for the purposes of this study we shall confine our analysis exclusively on UPDRS.

Speech disorders, which are of particular interest in this study, may be amongst the earliest PD onset indicators [12], and are reported in the vast majority of PWP [13]. Furthermore, strong empirical evidence has emerged associating speech performance degradation and PD symptom severity [12]–[17]. Recent work has highlighted the intrinsic link between speech and specific motor functionality in PD in terms of freezing [18], sensory impairment [19], and determining genetically-determined PD (through Leucine-Rich Repeat Kinase 2, LRRK2 mutations) [20].

Most PD studies rely on the use of expensive, purpose-built specialized hardware to record signals which are characteristic of PD symptoms, e.g. [9], [21]–[24]. We have previously demonstrated the considerable potential of speech to replicate the clinical scale UPDRS [9], [24]–[26], using high quality speech signals collected with Intel Corporation’s *At-Home Testing Device* (AHTD) [21]. This device collects high quality speech signals sampled at 24 kHz, following the established recommendation that a sampling frequency of at least 20 kHz should be used to extract clinically useful information [27].

In this study, we investigate whether it is possible to accurately infer UPDRS using speech signals transmitted over the standard cellular mobile voice telephone network, using a detailed simulation of the entire digital communication process. The rationale for using the existing voice mobile phone network over specialized, purpose-built hardware such as the AHTD is that (a) the existing voice network reaches nearly 75% of the global population, (b) economies of scale and global market competition has brought the price of access down so that it is affordable to a majority of the global population, (c) mobile telephony allows freedom of movement for PWP, eliminating the need to carry additional equipment when leaving home. Thus, the standard phone network provides convenient means towards inexpensive and frequent PD severity assessments, facilitating monitoring and potentially assisting rehabilitation. Data-mining of speech signals obtained using the public telephone network to extract clinically useful information has recently shown promising results [28]–[30]. Similarly, Saenz-Lechon *et al.* [31] investigated the effect of different data transmission rates in automatic voice pathology detection, and concluded that compressing signals (down to at most 64 kbps) does not prevent accurate detection of vocal pathologies.

We demonstrate that mobile phone technology could be useful in telemonitoring PD symptom severity, further endorsing previous findings that speech may offer a convenient framework for remote assessment [9], [24], [25].

## II. Data

We use the voice data collected by Goetz *et al.* [21], described in detail in Tsanas *et al.* [9]. In brief, 52 subjects with idiopathic PD diagnosis up to five years from the time of the baseline clinical visit were recruited into a clinical trial to investigate the potential of the AHTD. All subjects gave written informed consent, and did not receive PD-related treatment for the six-month duration of the trial. They were asked to complete a range of tests weekly during a convenient, pre-specified time window (all tests can be completed in about 20–30 minutes). Sustained vowel /ah:/ phonations, where the subject is asked to sustain vowel phonation at a comfortable pitch for as long and as steadily as possible, were part of the test protocol. Here we focus exclusively on these sustained phonations. Subjects were diagnosed with PD if they had at least two of the three hallmark PD symptoms (bradykinesia, rigidity, tremor), without evidence of other forms of Parkinsonism. We did not apply any exclusion criteria related to specific PD symptoms (e.g. depression). We disregarded data from 10 participants – two that dropped out of the study early, and from eight additional PWP that did not complete at least 20 valid study sessions during the trial period. Therefore, in this study we analyze data from 42 PWP.

Previously, we demonstrated that partitioning the data by gender is important in this application [9], [26], and hence males and females are studied separately here as well. The 28 male subjects were 64.8±8.1 (mean ±standard deviation) years old, with a PD diagnosis 63.0±61.9 weeks since diagnosis at trial baseline. Their motor-UPDRS scores were: baseline 20.3±8.5, three months into the trial 21.9±8.7, six months into the trial 22.0±9.2, and total-UPDRS scores were: baseline 27.5±11.6, three months into the trial 30.4±11.8, and six months into the trial 31.0±12.4. The 14 female subjects were 63.6±11.6 years old, with a PD diagnosis 89.7±81.2 weeks since diagnosis at trial baseline. Their motor-UPDRS was: baseline 17.6±7.4, three months into the trial 21.2±10.5, six months into the trial 20.1±9.4, and their total-UPDRS was: baseline 24.2±9.1, three months into the trial 27.4±12.1, and six months into the trial 26.8±10.8.

Six sustained vowel /ah:/ phonations were recorded each time the PD subject took the test: four at comfortable level of pitch and loudness, and two at twice the comfortable loudness (elicited with the instruction “twice as loud as the first time”). The signals were sampled at 24 kHz at 16 bit resolution. After initial processing to remove faulty phonations (e.g. patient coughing, failure to record phonation), we processed 4010 phonations for the male subjects, and 1865 phonations for the female subjects.

Although the phonations were recorded *weekly*, the actual clinical assessments for motor-UPDRS and total-UPDRS were obtained at trial baseline, three months into the trial, and at six months into the trial. To obtain weekly UPDRS estimates to associate with the phonations we used piecewise linear interpolation going exactly through the measured baseline, three-month and six-month UPDRS assessments [9], [24]–[26], [32]. This assertion builds on strong empirical evidence suggesting that *average* symptom progression in early PD stages (up to about five years) is almost linear in non-medicated patients as observed in clinical metrics [33], [34]. The PWP in the AHTD trial were in early PD stages (up to five years from disease diagnosis) and remained non-medicated for the duration of the trial, aspects which justify the use of piecewise linear interpolation when filling in missing data. The tacit assumption is that PD symptom severity did not fluctuate wildly within the intervals where the clinical scores were obtained. Discretizing the response variable to transform a regression problem into a classification problem is well known in the machine learning literature, and often this step can lead to better prediction performance. We have found that in this application it is better to discretize the interpolated UPDRS scores and work with *classifiers* instead of *regressors* [9], [35]; hence both motor-UPDRS and total-UPDRS were rounded to the closest integer value, giving rise to a multi-class classification setting. For another recent application from a different domain where this problem transformation was beneficial see [36].

For further details about the dataset and the AHTD data acquisition hardware, please refer to Tsanas *et al.* [9].

## III. Methods

We re-iterate that the aim of the study is to investigate UPDRS estimation using speech signals transmitted over the standard cellular mobile voice telephone network. Given that the data available in the study has been collected using the high quality AHTD equipment we have used a *digital communications simulation framework* to study the distorted signals received through a hostile data transmission channel. The following section describes in detail the process used to simulate the data transmission and reception process of the raw speech signals so that they resemble realistic distorted signals we may expect to have in a practical cellular mobile telephony network.

### A. Simulation of the Cellular Mobile Telephony Network

Creating a realistic simulation of the cellular voice telephony network requires the following steps: (a) encoding the AHTD speech signals into bit-streams for transmission, (b) simulating the transmitter, radio channel, and receiver, and (c) decoding the transmitted bit-streams back into intelligible speech recordings. This application requires only *one way* (simplex) communication; PWP call into an automated voice messaging service and leave sustained vowel phonations. Predictions of symptom severity are extracted from these voice messages and clinical personnel suggest the appropriate course of action offline as a result of the estimated UPDRS. Moreover, the sustained vowel phonations need only be a few seconds long, that is, considerably shorter in duration than most telephone conversations.

**FIGURE 1.**
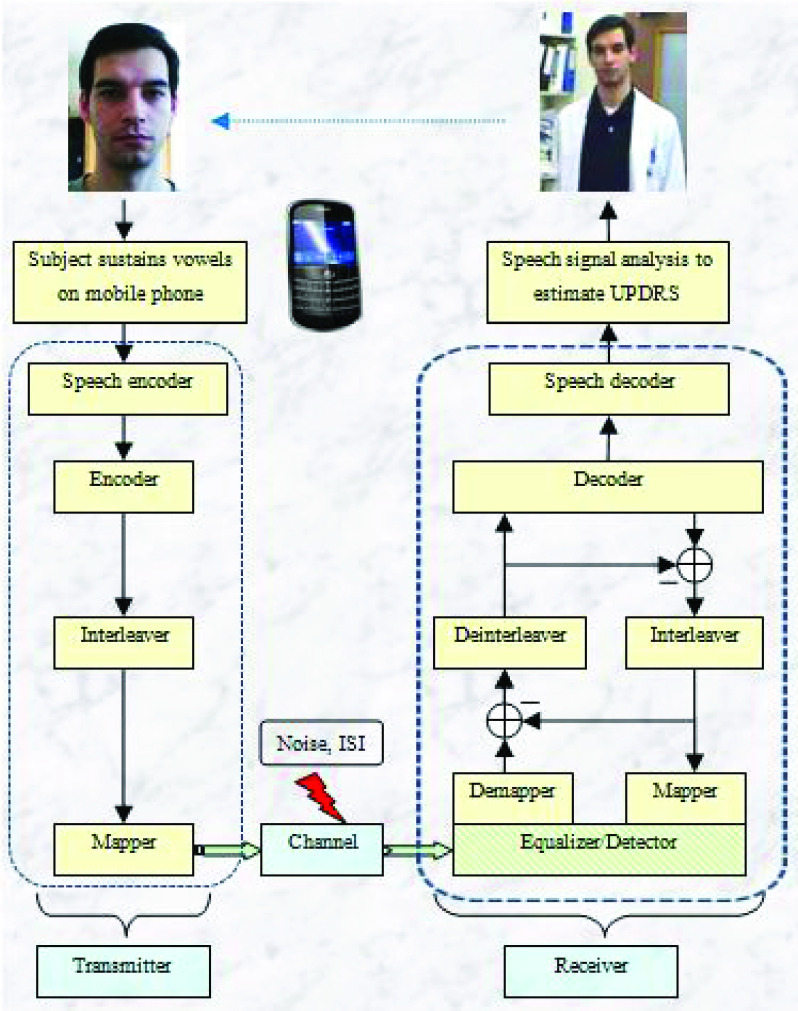
Schematic diagram of the digital communication process. ISI stands for Intersymbol Interference.

Fig. 1 presents the schematic diagram of the communication system used in this study. The main components of a digital communication system are the *transmitter*, the *channel* (physical medium connecting the transmitter and the receiver), and the *receiver*. The transmitter aims to assist the receiver to correctly recover the speech signal which may be distorted by the channel. We follow closely the studies of Tsanas [37], Ampeliotis and Berberidis [38], and Tuchler *et al.* [39] for the practical implementation. We summarize the data communications process in the Supplementary Material under section 1.1 ‘Overview of the data communication process’, and refer readers to specialized monographs for further background [40], [41].

### B. Methodology to Analyze the Speech Signals Recovered at the Receiver

We followed three steps to process the recovered phonations and extract clinically useful information: (a) *feature extraction*, where we applied speech signal processing algorithms to characterize the phonations and extract characteristic patterns (features), (b) *feature selection*, where a *parsimonious* (small, information-rich) subset of the originally computed features is selected in order to provide maximally useful information for predicting UPDRS, and (c) *feature mapping*, where a standard supervised learning algorithm was used to determine a functional form associating the selected feature subset with the clinical outcome (UPDRS). The rationale behind this methodology is that characteristic acoustic patterns in PWP’s voice are indicative of UPDRS. Although confounding factors may affect vocal performance (such as the subject’s emotional state, some pathological condition not related to PD, organic vocal pathology independent of PD, or pathologies due to tobacco abuse), it is unlikely these contaminate more than a handful of the approximately 6000 recordings used here. We assumed that potential confounding factors do not dominate PWP’s voices to the extent that the extracted dysphonia measures do not provide clinically useful information for estimating UPDRS.

Before characterizing the phonations by extracting dysphonia measures, we removed the vowel onset and offset choosing the three seconds in the middle of the phonation to simplify computational processing. The resulting three second signal was subsequently normalized to facilitate comparisons across recordings.

#### 1) Feature Extraction

We applied the dysphonia measures rigorously defined in Tsanas *et al.* [9] to the speech signals recovered at the receiver. We refer to that paper for detailed description of the concepts and rationale behind each algorithm. The MATLAB source code to compute these features is available on the first author’s website (https://www.darth-group.com/software). Here, we briefly describe the most important families of dysphonia measures used in this and other studies.

Some of the most widely used dysphonia measures are *jitter* and *shimmer* [27], [42]. They seek to capture the physiological observation that the vocal fold vibration pattern is nearly periodic in healthy voices, whilst it is disturbed in pathological voices [42]. Jitter characterizes deviations in fundamental frequency (F0), whereas shimmer characterizes deviations in amplitude. There is no unique definition of those dysphonia measures, and we investigated many *jitter* and *shimmer variants* [15] which are algorithmic variations of the same underlying concept. Quantifying vocal fold departure from near periodicity has inspired the development of the *Recurrence Period Density Entropy* (RPDE) [43], the *Pitch Period Entropy* (PPE) [44], the *Glottal Quotient* (GQ) [9], and F0-related measures [9]. GQ can be seen as an improved jitter-like family of measures, but working directly with vocal fold cycles instead of pre-specified segments (e.g. 10 ms) of the speech signal. RPDE expresses the uncertainty in vocal fold cycle duration. PPE quantifies the impaired control of F0 in sustained phonations, taking into account normal vibrato. The F0-related measures include statistical summaries of F0 distributions, and F0 differences compared to average age- and gender-matched healthy controls in the population.

The second group of dysphonia measures characterize *Signal to Noise Ratio* (SNR)-like quantities. The physiological motivation for this group is that incomplete vocal fold closure leads to the creation of aerodynamic vortices which result in increased acoustic noise. *Harmonic to Noise Ratio* (HNR) [42], *Detrended Fluctuation Analysis* (DFA) [43], *Glottal to Noise Excitation* (GNE) [45], *Vocal Fold Excitation Ratio* (VFER) [9], and *Empirical Mode Decomposition Excitation Ratio* (EMD-ER) [9] are typical examples. GNE and VFER analyze the frequency ranges of the signal in bands of 500 Hz. Empirically, we found that frequencies below 2.5 kHz can be treated as ‘signal’, and everything above 2.5 kHz can be treated as ‘noise’ [9], [35] to define SNR measures using energy, nonlinear energy (Teager-Kaiser energy operator) and entropy concepts. EMD-ER is similarly motivated: the Hilbert-Huang transform [46] decomposes the original signal into its constituent components in decreasing order of contributing frequency. Then, the top (high frequency) components are taken to constitute noise, and the lower frequency components to constitute signal, to obtain SNR-like measures.

Lastly, *Mel Frequency Cepstral Coefficients* (MFCC) have been traditionally used in speaker recognition applications, but also appear promising in biomedical speech signal processing contexts [9], [35], [47], [48]. Although the participants in this study were asked to sustain a vowel (hence theoretically the vocal folds have a steady oscillating pattern and the vocal tract remains completely steady), it is reasonable to argue that the articulators will exhibit some perturbation (similarly to the fact that the vocal folds will not vibrate with perfect periodicity, even for healthy controls when sustaining a vowel [27]). The MFCCs collectively characterize the short-term power spectrum of a speech signal on the nonlinear (Mel) scale, which approximates the human auditory system’s response more closely than the linearly-spaced frequency bands. Thus, they inherently quantify the filtering effects of the vocal tract (if we consider the conceptually appealing source-filter voice production mechanism [27]). Therefore, MFCCs can be considered to detect subtle changes in the position and motion of the articulators (tongue, lips) which are known to be affected in PD [13].

Overall, we applied 132 dysphonia measures to the speech database, each dysphonia measure producing a single real value per voice sample, resulting in a *design matrix* of size $4010\times 132$ for male PWP and a matrix of size $1865\times 132$ for female PWP.

#### 2) Feature Selection

The use of a large number of features (132 in this study) makes it extremely difficult to discern meaningful patterns in the data, and may often be detrimental in the process of mapping the features onto the clinical outcome UPDRS. This problem is known as the *curse of dimensionality*, and arises because adequate population of the feature space requires that the number of voice samples increases exponentially with the number of features [49]. Contemporary algorithms that can map features onto outcomes may be very robust to the inclusion of potentially noisy or irrelevant features, and their predictive power may or may not be severely affected; however, a smaller feature set always facilitates insight into the problem by allowing interpretation of the most predictive features [50], [51]. An exhaustive search through all possible feature subset combinations is computationally impractical; *feature selection* (FS) algorithms are a principled approach to selecting a smaller (lower dimensional) feature subset. We refer to Guyon *et al.* [51] for a detailed overview of FS.

Here, we compared four FS algorithms: (1) *Least Absolute Shrinkage and Selection Operator* (LASSO) [52], (2) *Minimum Redundancy Maximum Relevance* (mRMR) [53], (3) *RELIEF* [54], and (4) *feature importance* in Random Forests (RF) [55]. We applied the FS voting strategy that was previously described in Tsanas *et al.* [35], [48], [56] to identify the final feature subset $S$ for each FS algorithm, which was used in the subsequent statistical mapping phase. We refer readers to section 1.2 ‘Background on feature selection’ of the Supplementary Material for further background on FS and the FS voting strategy.

#### 3) Feature Mapping

In the preceding steps we have computed 132 characteristic patterns from the sustained vowel phonations, and subsequently applied FS techniques to obtain subsets of those features. Here, we aim to determine the functional relationship $f\left ({\mathbf {X} }\right)=\mathbf {y}$, which maps the dysphonia measures $\mathbf {X}=\left ({\mathbf {x}_{1}\ldots \mathbf {x}_{M} }\right)$, where $M$ is the number of features, to the outcome (response) **y** (motor-UPDRS and total-UPDRS in this study). That is, we want to obtain a *classifier* that will use the dysphonia measures to accurately predict UPDRS. There is a large literature on supervised classification, and we refer to Bishop [49], and Hastie *et al.* [50] for a broad overview of this area. Here, we experimented with three powerful classifiers: Random Forests (RF), Support Vector Machines (SVM), and eXtreme Gradient Boosting (XGBoost). For more specific background on these statistical learners please see section 1.3 ‘Background on statistical learners’ in the Supplementary Material.

#### 4) Model Validation and Generalization

As in previous studies [9], [24], [26] we used 10-fold Cross Validation (CV) to assess the generalization performance of the statistical learners. Conceptually, CV provides an estimate of the accuracy with which UPDRS may be predicted on a new dataset, assuming the new dataset has similar statistical characteristics to the data used to train the classifier. Specifically, we split the initial dataset comprising $N$ (4010 for males and 1865 for females) phonations into a training (in sample) subset of $0.9\cdot N$ (3609 and 1679) phonations and a testing (out of sample) subset of $0.1\cdot N$ (401 and 186) phonations. For statistical confidence, the process was repeated a total of 100 times, randomly permuting the data each time before splitting into training and testing subsets. As in previous studies [9], [24]–[26], we used the Mean Absolute Error (MAE) to assess the model performance:

\begin{equation*} \mathrm {MAE}=\frac {1}{N}\sum \nolimits _{i\in Q} \left |{ {\hat {y}_{i}-y}_{i} }\right |\tag{1}\end{equation*}

where $\hat {y}_{i}$ is the predicted UPDRS and $y_{i}$ is the actual UPDRS for the $i^{\mathrm {th}}$ entry in the training or testing subset, $N$ is the number of phonations in the training or testing subset, and $Q$ contains the indices of that set. Errors over the 100 CV iterations were averaged. We also computed the Confidence Interval (CI) of the errors (using 95% confidence level).

Finally, we also trained and assessed the model performance by using a validation scheme leaving samples out from a participant. Specifically, the data from the $L$-1 participants (where $L$ is the total number of subjects, 28 males and 14 females for the models we build, respectively), the data from the first four weeks of the left-out participant were used for training, and the model performance was reported for the remaining five months. In addition to the dysphonia measures we presented RF with the UPDRS values during the first four weeks for the left-out participant (this would be known in practical setting in this tracking scenario and is implicitly a calibration approach). This model validation approach replicates the tracking setting where past data from the same subject in addition to the database built from the remaining $L$-1 participants are used to replicate future UPDRS scores for each of the participants. Errors for the weekly UPDRS scores were averaged. We did not include timing information, participants’ age, or participant identifiers as inputs into the statistical learners in order not to implicitly bias the statistical learning models.

## IV. Results

**FIGURE 2.**
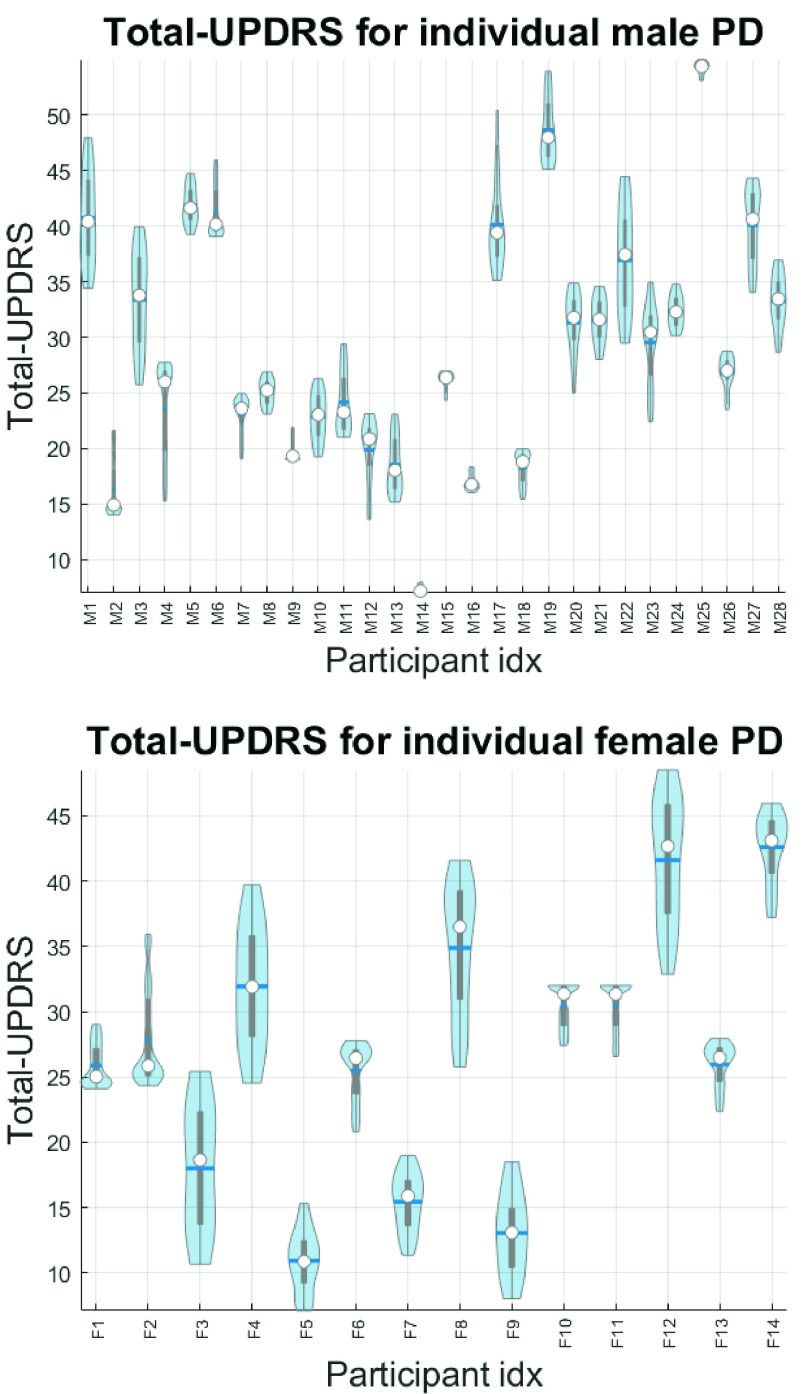
Violin plots with the total-UPDRS variability within each of the 28 male participants and 14 female participants in the study. The white dot in each violin indicates the median, the grey box represents the range for the 25^th^ percentile (bottom) and 75^th^ percentile (top) entries. The horizontal line indicates the mean value.

**FIGURE 3.**
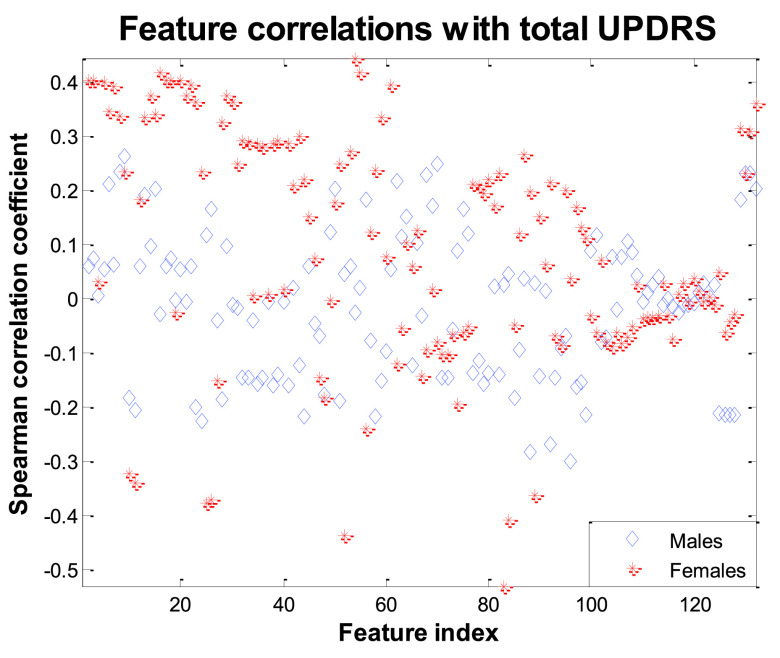
Visual representation of the feature correlations with total UPDRS. Features 1 to 30 are jitter variants, 31–51 shimmer variants, 51–60 HNR and GQ variants, 59–82 energy-related measures (RPDE, GNE, EMD-ER), 83–124 MFCCs, 125–132 F0-related features.

Prior to any analysis, it is useful to visually appreciate the variability of UPDRS within participants. Fig. 2 presents violin plots with the total-UPDRS variability for each participant in the study, stratifying the data by sex. We clarify that we used all weekly UPDRS estimates derived using linear interpolation to present here (rather only the three UPDRS clinical assessments per participant) because these are subsequently used as the ground truth for training and testing the statistical learners. We remark that for some participants the UPDRS range is over 10 points (7.67 ± 4.12 for males and 9.69 ± 4.22 for females). We computed Spearman correlation coefficients to quantify the strength of statistical association of the features with UPDRS, and compared these new association strengths to our previous findings [9] (see Tables 1 and 2 for comparisons of the correlation coefficients of indicative features with UPDRS for males and females, respectively). The results in these two tables illustrate the changes in the univariate statistical association of the features with UPDRS and implicitly demonstrate the effect the noise and the data transmission channel have in terms of using speech signals to replicate PD symptom severity. As expected, in most cases there are stronger statistical associations with the raw data; there are a few cases where the magnitude of the correlation coefficients appears slightly larger in the noisy data which can be attributed to statistical fluctuations. We have found that, as expected, the features in the present study had lower association strength with motor-UPDRS and total-UPDRS than in earlier studies that used full bandwidth speech [9]. Fig. 3 provides a succinct representation of the univariate association of each feature with total-UPDRS. Overall, univariate associations appear to be stronger for females, particularly for features which focus on F0 (jitter, GQ, F0-related features). We report the out-of-sample accuracy (using RF) with which UPDRS can be predicted in Supplementary Material Table 1 for males, and Supplementary Material Table 2 for females. For each FS algorithm, the final number of features $K$ is determined using the *one standard error rule* [50]: adhering to the principle of parsimony, we fix $K$ to be the number of features where MAE is up to one standard deviation larger than the globally lowest MAE obtained with the feature subsets from that FS algorithm. The MAE for motor-UPDRS is 2.91 ± 0.23 (CI=[2.49, 3.46]) for males and 2.38 ± 0.23 (CI=[2.19, 3.13]) for females, whilst the MAE for total-UPDRS is 3.43 ± 0.27 (CI=[3.08, 4.11]) for males and 2.91 ± 0.27 (CI=[2.58, 3.54]) for females. The out-of-sample performances using SVMs and XGBoost are not presented because results were consistently worse compared to RF.

**TABLE 1:** Correlation Coefficients of Features With Total UPDRS in Males (Extracted From the Raw Data and From the Noisy Data)

Feature name	Spearman correlation coefficient R	
	Raw data	Noisy data
Jitter (abs 0^th^ perturbation)	0.12	0.12
Shimmer (abs differences)	−0.14	−0.02
HNR	−0.02	0.05
GNE_NSR,TKEO_	0.11	0.10
DFA	−0.21	−0.10
PPE	0.03	0.05
VFER_NSR,entropy_	0.19	0.16
Log energy	0.17	0.03
6^th^ MFCC	−0.29	−0.15
Std F0	0.15	0.18

In all cases the features were statistically significantly (p<0.05) correlated with UPDRS. We present one indicative feature from each algorithmic family of dysphonia measures.

**TABLE 2:** Correlation Coefficients of Features With Total UPDRS in Females (Extracted From the Raw Data and From the Noisy Data)

Feature name	Spearman correlation coefficient R	
	Raw data	Noisy data
Jitter (abs 0^th^ perturbation)	0.38	0.34
Shimmer (abs differences)	0.33	0.25
HNR	−0.44	−0.44
GNE_NSR,TKEO_	0.12	0.12
DFA	−0.02	0.07
PPE	0.40	0.39
VFER_SNR,SEO_	−0.18	−0.07
Log energy	−0.49	−0.53
12^th^ MFCC	0.26	0.04
Std F0	0.32	0.31

In all cases the features were statistically significantly (p<0.05) correlated with UPDRS. We present one indicative feature from each algorithmic family of dysphonia measures.

The methodology was repeated contaminating the speech signals with AWGN or pink noise prior to speech coding and transmission. In both cases, the results were very similar (slight differences due to statistical fluctuation). By comparison, in a recent study in this application where high-quality, high-bandwidth, uncompressed speech signals from the AHTD were used instead, the MAE reported for total-UPDRS was [26]: 1.49 ± 0.14 for males, and 2.14 ± 0.25 for females. We defer further elaboration of those findings for the Discussion.

Finally, for the model validation approach where we used the data from $L$-1 participants and only the first month of the data for each left-out participant to train the model and aiming to estimate their future total-UPDRS in the following 5 months (UPDRS tracking), we obtained MAE 3.72 ± 2.29 (CI=[2.83, 4.47]) for males and 4.86 ± 2.42 (CI=[3.67, 5.86]) for females.

## V. Discussion

We had previously demonstrated that using speech signals may be very promising in both (a) differentiating PD subjects from age- and gender-matched healthy controls [48], and (b) telemonitoring PD symptom severity by means of replicating the standard clinical scale UPDRS [9], [24]–[26]. In all those studies we had used high-quality speech signals, collected using high sampling frequency with minimal signal distortion (for example the signals were collected in a sound-treated booth in [48]). In this study, we investigated the robustness of using lower quality signals which have been transmitted through the simulated GSM mobile telephone network. We found strong evidence that the existing GSM network, which to-date reaches more than 5 billion subscribers, enables clinically accurate UPDRS estimation.

In Tsanas *et al.* [26], where the high-quality signals obtained from Intel’s AHTD were used, we reported that UPDRS could be estimated to within 1.5 UPDRS points for males and 2.2 UPDRS points for females; here we demonstrated that UPDRS can be estimated to within approximately 3.4 UPDRS points for males, and 2.9 UPDRS points for females (when comparing results against [26] where also 10-fold CV was used). We argue that this loss in accuracy of UPDRS, which is due to bandwidth restriction and/or channel transmission error is acceptable in practice because most PWP who could benefit from remote symptom tracking, are unlikely to have access to expensive, dedicated hardware such as the AHTD. We emphasize that the accuracy with which UPDRS is estimated even in this scenario of restricted quality speech, is less than the *inter-rater variability* (difference in UPDRS score between two expert clinicians), which is about 4–5 points [57]. Putting our findings in the wider context: clinical colleagues had previously remarked that in their view our early investigations in 2010 towards replicating UPDRS using speech were insufficiently accurate to be widely deployed in clinical practice (the MAE in that study was 7.5 points) [24]. They had emphasized this technology would be practically very useful if we could demonstrate the MAE to be better than the inter-rater variability (i.e. less than 5 UPDRS points). This has been the informal threshold that we had used as guidance to deem whether our findings are practically ‘sufficiently good’. Therefore, speech over GSM remains clinically useful here, and could be used as a decision support tool to aid clinicians in remote, non-invasive PD symptom severity assessment. Similarly, the automatic assessment of voice pathologies using signals transmitted over the public telephone network had been shown to be promising in related applications [28], [29].

The topic of the appropriate methodology towards reporting out-of-sample performance is considerably more subtle than it first appears and has attracted some recent attention [58], [59]. The latter article contains discussion from three research groups weighing on the topic of how best to provide an estimate of generalization performance in clinical settings. We remark that the first guiding principle in deciding on the model validation scheme is how we envisage the deployed model will be subsequently used in practice (i.e. the intended usage should dictate the model validation approach). The argument is that standard CV may include confounding variables which could potentially overestimate performance because samples from the same subject end up in both the training and testing subsets [58]. In the debate appearing in [59] there is discussion and different opinions on confounders and which approach should be used when aiming to develop a tool towards *diagnosis*. However, all three research teams essentially agree that standard leave-subject-out methods underfit the data when it comes to *tracking* and endorse the use of validation methods where samples from the same subject are used in both training and testing sets. In particular, Varoquaux recommends only using samples from the subject’s past to estimate future entries in this tracking setting [59]. We emphasize that the problem investigated in this study comes under the broad area described as tracking in the studies above [58], [59]. Motivated by these points, we have introduced a model validation approach where we used data from the first 4 weeks of a participant in the training set (in addition to the data from all other $L$-1 participants), and tested the performance on the remaining five months for the left-out participant. We demonstrated that also in this case we have relatively accurate results (MAE for total-UPDRS is 3.72 ± 2.29 for males and 4.86 ± 2.42 for females). The MAE is lower in males, likely reflecting the lower average individual UPDRS variability observed in Fig. 2. From a practical perspective, this model validation approach we used here is directly comparable to a tracking paradigm that records some phonations and obtains the UPDRS clinical assessments by an expert neurologist for a specific participant before deploying the tool for longer-term UPDRS tracking.

In a related earlier study, Bayestehtashk *et al.* enrolled 168 PD participants and focused on replicating motor-UPDRS using sustained vowels /ah/, a diadochokinetic task, and a reading task (using standardized, linguistically rich text) [60]. They reported a MAE of 5.5 motor-UPDRS points. To the best of our knowledge, this is the only study by a different research group that used speech to replicate symptom severity as expressed using UPDRS and quantitatively expressed performance using some error metric.

Concurring with previous findings, we have found that a parsimonious speech feature subset actually improves the out-of-sample MAE, and is also more amenable to interpretation [9], [24], [26]. We experimented with different statistical learners aiming to improve the out of sample performance. In addition to RF, we explored SVMs (linear SVM, polynomial SVM), and XGBoost (both in regression and classification mode), exploring different configurations and optimization of their internal hyper-parameters (results not shown). The radial basis function SVM was considerably better than linear SVM and generally better than the polynomial SVM. RF outperformed SVMs and XGBoost consistently and significantly ($p < 0.001$), although we cannot provide a clear theoretical justification for this finding. More detailed empirical and theoretical analysis is required to understand which classification algorithm is likely to lead to more accurate prediction for similar datasets [35].

The UPDRS scores used as the response variable in our investigations have different class membership, i.e. this is an unbalanced multi-class classification statistical learning setting, which is known to be challenging in practice. There are different strategies to cope with the class unbalance problem including (a) using different weights internally in the classifier for the samples belonging to different classes (as a function of sample domination in the training data in each iteration, where under-represented classes are up-weighted), and (b) using different probabilistic cut-offs for the different classes (again, these can be set to be inversely proportional to class dominance). We stress that these adaptive thresholds should only use information from the training dataset (similarly, in a CV application these need to be recomputed accordingly using only the information available in the training of the classifier). We have explored both approaches to train different RF models, however neither led to improving the out of sample performance (detailed results not shown). There is a more sophisticated approach to tackle class unbalance in statistical learning, by generating new (artificial) data points, e.g. using techniques such as the Synthetic Minority Over-sampling Technique (SMOTE) [61] and Adaptive Synthetic Sampling (ADASYN) [62]. Then, we can explore using the augmented dataset (comprising both the original and artificial data) in the statistical learning process. Again, there are different strategies within data generation, e.g. to ensure all classes have equal membership (completely balanced dataset), or ensuring there is no clear dominance of particular class(es) in order not to contaminate the data with a very large number of artificial samples. We had not explored these data generation approaches here because it would diverge from the main focus of the study, however it would be an interesting direction to pursue in further work.

We used four FS algorithms and applied a voting mechanism approach across perturbed versions of the dataset (see Supplementary Material for details, including the discussion therein on FS strategies) to identify features which are jointly most predictive of UPDRS. The use of perturbed versions of the data enables the investigation of consistency *within* each FS algorithm, and the use of different FS algorithms provides insight into FS consistency *across* FS algorithms. The non-classical dysphonia measures (mainly IMF$_{\mathrm {NSR,SEO}}$, VFER) and MFCCs are consistently selected as the most predictive features by RELIEF and RF feature importance (which appear to lead to the lowest MAE, see Supplementary Material Tables 1 and 2). The selection of MFCCs is very consistent across all FS algorithms. These results reinforce previous findings suggesting that dysphonia measures focusing on energy aspects may be promising for vocal pathology assessment [35], [48]. Similarly to previous studies [9], [26], [35], the features selected are gender-dependent and focus on different pathological effects in PWP’s voice. This finding supports the tentative physiological suggestion that the underlying processes of degradation in PD speech may be different in men and women [9], [35]. For females the selected features are mainly log energy, low MFCCs and F0 related measures; for males they are DFA, IMF$_{\mathrm {SNR,SEO}}$, VFER$_{\mathrm {NSR,entropy}}$, and mid-range MFCCs. Overall, the most promising characteristic in PD speech pathology for males appears to be working with energy in the higher energy bands: quantifying stochastic turbulent noise (DFA), excitation of different frequency bands and turbulent noise in vocal fold cycles (VFER), and ratio of high frequency (>2.5 kHz, denoting ‘noise’ in the signal) over low frequency (< 2.5 kHz) energy (IMF). For females, the most promising characteristic in PD speech pathology appears to be the signal energy (log energy, 0th MFCC). Interestingly, some dysphonia measures that rely on F0 may also provide clinical information for females but not for males. As we argued previously [9], [35], this finding may be because natural male voices have considerably more vibrato (physiological tremor) compared to female voices. Given that females have higher F0 on average [27], and that higher F0 is normally associated with lower F0 variability [42], F0 perturbations might reflect voice pathology in females whilst similar distortions in males’ vocal performance could be, at least partly, attributed to normal vibrato. This is likely the same underlying reason why log energy is very strongly associated with UPDRS in females ($\text {R}={-}0.53$) but poorly associated with UPDRS in males: log energy captures the main ‘power’ in the signal which is primarily driven by the contribution of the lungs and vocal folds (as the source of the recorded signal, considering the basic source-filter model of the vocal production mechanism [27]). It is possible that the vocal folds in PD might be more strongly affected in females compared to males, and also normal vibrato in males might be masking underlying F0 perturbations (which in female voices may more clearly indicate underlying pathology) [35]. Incidentally, the negative correlation of log energy with UPDRS verifies what is intuitively expected: reduced log energy corresponds to reduced loudness (which is well reported in PD [13]) and may be used as a marker of symptom severity.

MFCCs have been widely used in speech applications and have been previously shown to perform very well in related biomedical applications, e.g. [47]. This study further supports their use as powerful features in PD monitoring, as evidenced in the FS findings reported in Supplementary Material Tables 1 and 2. Although MFCCs are well-founded from a speech signal processing perspective, their physiological interpretation is more challenging. The lower MFCCs reflect the amplitude and envelope spectral fluctuations, and higher MFCCs convey mainly information about harmonic components; mid-range MFCCs are not easily interpretable.

We had previously reported that the VFER family of dysphonia measures is amongst the best approaches to quantify information in speech signals to estimate UPDRS for males [9], [35. Although VFER measures were still selected here by all FS algorithms, they do not appear near the top of RELIEF and RF feature importance. This may be because VFER relies on quantifying the information in the high frequencies (>2.5 kHz) as ‘noise’; however due to the reduced bandwidth when using a sampling rate of 8 kHz much of this high-frequency information is lost. This would suggest that the effectiveness of VFER relies on using high sampling rates (>20 kHz), in order to accurately quantify the extent of high frequency noise in the signal. In general, young adult pathology-free voices may be harmonically efficient up to about 6 kHz; therefore the suggested threshold of 2.5 kHz for denoting ‘noise’ may require further clarification. This empirical finding was reported in Tsanas *et al.* [9], considering frequencies below 2.5 kHz to denote ‘signal’ and frequencies above 2.5 kHz to denote ‘noise’: the threshold was optimized scanning frequencies (using steps of 500 Hz similarly to Michaelis *et al.* [45]) in order to determine UPDRS. Interestingly, broadly similar findings regarding the threshold of ‘signal’ and ‘noise’ have been described by other research groups. For example, Gomez-Vilda *et al.* [63] indicated that frequencies above 2 kHz can be generally considered turbulent noise. Likewise, the Multi-Dimensional Voice Program (MDVP - http://www.kayelemetrics.com/) program includes “Voice Turbulence Index”, which is an alternative dysphonia measure relying on the SNR concept, where the spectral energy above 2.8 kHz is used to denote the high frequency energy component in the speech signal [64]. Overall, we tentatively suggest that the empirical 2.5 kHz threshold may have a solid physiological justification which is reflected broadly in the findings of different researchers: most of the energy in the sustained vowels is up to the second formant, and the second formant can be up to about 1.7 kHz for the sustained vowel /ah:/ [27].

One very interesting new finding in this study is that UPDRS estimation in males deteriorates considerably more compared to UPDRS estimation in females as a result of the lower quality speech signals. This may be related to the bandwidth restriction, but may also be a consequence of the finite bit allocation available to reproducing the pitch period with pitch pulses. It could also be due to the increased noise that is masked by the formants in the perceptually-weighted linear prediction: this noise may not be heard, but may, nonetheless, be important in PD.

The future of health telemonitoring is linked to the potential of smartphones and associated apps. A promising development in that direction would be the deployment of a smartphone app that can record high-quality (wide-band and low-distortion) speech signals. This further underlines the generalization potential of using speech signals towards future PD symptom monitoring systems. Nevertheless, there are still many people (particularly elderly, who are the main beneficiaries of the proposed technology) that do not own or do not know how to operate a smartphone. Although it is conceivable this might change in the next 10–20 years as smartphones are becoming more affordable and the current generation of 50–60 year-old people are generally better adapted to the use of smartphones, we envisage the proposed technology here with standard mobile telephony may remain pertinent because of its simplicity in use.

Our findings confirm the established view in the clinical speech community suggesting that speech signals of at least 20 kHz should be preferred in clinical applications because there is useful information in the higher frequencies of the spectrum [27]. Nevertheless, the performance degradation as a result of the use of the lower-quality GSM coding and communication framework is unlikely to be prohibitive for clinically useful UPDRS prediction. We conjecture that this may also be the case for other voice pathologies. We hypothesize that the speech community may have, hitherto, been overly pessimistic in the need for very high-quality speech signals [27] in clinical speech science.

We stress that the results reported in this study were obtained in a simulated digital communications framework involving the GSM standard. Additional tests in real-world contexts using actual mobile phones would be required to validate the robustness of the presented methodology. For example, in practice the channel may or may not always introduce additive white Gaussian noise, although this is generally the assumption in the digital communication literature [40], [41]. Also, we have not simulated the effect of drop-outs due to cell handoff, or switching between 2G/3G, or quality reduction due to the user not placing the phone close to their mouth. For this reason, our channel is chosen to be extremely noisy which introduces quite severe speech signal quality degradation. Additional factors that are hard to control, such as the mobile phone’s microphone, might need to be taken into account in a real application. Ideally, the microphone should exhibit uniform frequency response over the frequencies of interest (50 Hz – 10 kHz) to minimize spectral distortion. Similarly, microphones with reasonable SNR (>50 dB) may be required to ensure sufficient recording quality. Most commercially available microphones embedded in mobile phones adhere to these requirements. A detailed comparison of different microphones would reveal the extent to which speech signals are affected, and whether additional processing is necessary for signals recorded using mobile phones. Similarly, we have not pursued a full-scale simulation of different SNR and communication channels because this would involve reporting error rates and repeating the entire process with feature extraction, selection, and statistical mapping for different simulation scenarios. Instead we chose a moderate SNR (10dB) and a particularly hostile environment with the Proakis C channel which is frequently used in the literature to assess the performance of simulated digital communication approaches [40]. Therefore, we are reasonably confident that this study reports findings on a very challenging simulated digital communications environment.

It is not straightforward to test the proposed methodology in practice: this would involve building the receiver block and ideally testing (a) several types of commercially available microphones and analogue-to-digital conversion hardware, and also (b) the reception of signals in various realistic scenarios (e.g. in a rural, urban, or hilly environment), which would probably introduce additional distortion to the transmitted signal. Other scenarios to test include cell handover. Development engineers would need to test the proposed methodology in such diverse practical settings in future work. As we reported recently through a systematic review, there are relatively few paradigms where research findings are translated into digital health interventions to benefit patients [65]. The promising findings presented in this study and the reported results by other colleagues provide compelling evidence to suggest this is a sufficiently mature field to merit detailed testing in a new study that will explore all these different practical challenges.

The research area of speech signal processing and PD has generated considerable interest in the scientific community in the last 10–15 years and has led to some recent exciting developments. For example, there has been consistent interest in the binary differentiation of PD from healthy controls [48], [66], [67] amongst different research teams, which has generally led to very successful outcomes. We have recently reported on our findings in the Parkinson’s Voice Initiative (PVI), a large international study where we had collected more than 19,000 sustained vowel /a/ phonations across seven countries [30]. The PVI phonations were collected under acoustically non-controlled conditions over the phone with the explicit aim of investigating large scale population screening towards PD assessment using telephone-quality speech. We had demonstrated clinically meaningful differentiation of PWP versus controls, thus highlighting the potential of this technology at scale. Recently, researchers have started exploring speech data from different corpora which may lead to new insights across PD populations with different linguistic backgrounds [30], [68]. Furthermore, some studies have investigated different speech tasks for PD evaluations [60], [68], although it is still early to decide whether any particular task is clearly better that competing approaches. Another area of recent interest is in terms of associating acoustic features with clinical interventions, e.g. with pharmacological treatment (L-dopa) [69] and deep brain stimulation [70]. Collectively, these studies highlight the enormous potential of speech signal analysis in diverse PD areas. Moreover, we are currently collecting longitudinal data (including speech) from a large number of people at risk of PD, aiming to retrospectively revisit data from those people who are subsequently clinically diagnosed with PD. This would help us potentially develop a tool towards PD prognosis.

Telemonitoring in healthcare has received considerable attention lately, but global adoption is always constrained by the prohibitive costs associated with specialized telemonitoring hardware or equipment. Indicative recent explorative applications in the PD domain include the mPower study using iPhones to record a series of motor and cognitive tasks [71], and other studies relying on the capabilities of smartphones [72], [73], and wearables [74], which are not necessarily affordable and accessible to elderly PD patients leaving in rural areas. The exploration of highly cost-effective solutions, such as exploitation of existing cellular or PSTN telephone networks investigated in this study may be a critical step towards more widespread diffusion of this promising technology. We envisage the results of this study being a first step towards practical, affordable, and accurate telemonitoring of PD for the population at large.
